# Characterization of Post-COVID-19 Clinical Manifestations Among Patients Visiting a Post-COVID-19 Clinic in a Tertiary Care Center: A Descriptive Study

**DOI:** 10.7759/cureus.41523

**Published:** 2023-07-07

**Authors:** Sivaselvi C, Manju Rajaram, Jayalakshmi Ramakrishnan, Vishnukanth Govindaraj, Vemuri Mahesh Babu, Subathra Adithan, Mukta Wyawahare

**Affiliations:** 1 Pulmonary Medicine, Jawaharlal Institute of Postgraduate Medical Education and Research, Puducherry, IND; 2 Preventive Medicine, Jawaharlal Institute of Postgraduate Medical Education and Research, Puducherry, IND; 3 Pulmonary Medicine, All India Institute of Medical Sciences, Bibinagar, Puducherry, IND; 4 Radiodiagnosis, Jawaharlal Institute of Postgraduate Medical Education and Research, Puducherry, IND; 5 General Medicine, Jawaharlal Institute of Postgraduate Medical Education and Research, Puducherry, IND

**Keywords:** post-covid symptoms, post-covid-19, sars-cov-2, hospitalization, clinical manifestations, covid-19

## Abstract

Background

On December 21, 2019, a pneumonia-like outbreak of an unknown cause or origin was found to be emerging in Wuhan, China. In India, the first case of COVID-19 was found in Kerala and then started to spread all over India. Most of the infected people have recovered from the disease, but some patients were found to have symptoms at post-discharge follow-up. Although there are many studies on COVID-19 symptoms and signs during hospital stays, there is a scarcity of information regarding post-COVID-19 manifestations. The purpose of this study is to analyze the clinical characteristics of post-COVID-19 symptoms in patients attending the post-COVID-19 clinics.

Methods

A descriptive study was started on August 2021 at a tertiary care hospital in Southern India after institutional research and ethics committee clearance. All patients attending the post-COVID clinic, who tested positive for COVID-19 (RT-PCR or rapid antigen test (RAT) diagnosed or radiographically diagnosed COVID-19 (COVID-19 Reporting and Data System [CO-RADS] 5) were recruited. The proportion of people developing post-COVID-19 manifestations and categorization of symptoms in post-COVID-19 and its relationship to the severity of COVID-19 infections and the differences in post-COVID symptoms between hospitalized and non-hospitalized patients were studied.

Results

We enrolled 227 post-COVID patients who presented to the post-COVID clinic. The median age (IQR) of the participant was 52 (38-59) years, and the male-to-female ratio was 126/101 (1.24). Among 227 patients, 164 (72%) patients had exertional dyspnea, 109 (48%) patients had cough with expectoration, 96 (42.2%) patients with fatigue, 28 (12.33%) patients had myalgia, 18 (7.92) patients had a fever, 12 (5.28%) patients had hair loss, and 30 (13%) had other issues (loss of smell, sleep disturbances, and headache). Among 227, 142 (62.5%) patients were admitted to the hospital for acute COVID-19, and 85 (37.4) patients were in home isolation, but no statistical significance was found between in symptoms.

Conclusion

From this descriptive study, a high prevalence of post-COVID symptoms was noted, such aslike post-SARS syndrome. Mostly, researchers and clinicians have focused on acute COVID-19, but long-term follow-up with multidisciplinary evaluation and treatment is needed in all patients who recovered from acute COVID-19.

## Introduction

On December 21, 2019, a pneumonia-like outbreak of an unknown cause or origin was found to be emerging in Wuhan, China. On March 11, 2020, the World Health Organization declared the COVID-19 outbreak a pandemic. The virus that causes coronavirus disease 2019 (COVID-19) is designated severe acute respiratory syndrome coronavirus 2 (SARS-CoV-2) [1]. Clinical presentations of COVID-19 infection may vary from asymptomatic to multiorgan failure. Severe infection can lead to complications including pneumonia, blood clotting, myocarditis, acute myocardial infarction, acute kidney injury, acute respiratory distress syndrome (ARDS), sepsis, multiple organ failure, and other viral and bacterial infections that are not unique to coronavirus. Multiorgan involvement in COVID-19 may be because of the multiorgan tropism property of the angiotensin-converting enzyme receptor [2]. Disease severity was related to age and comorbidities of the infected individual. The COVID-19 symptoms last for an average of 11.5±5.7 days [3]. Most of the COVID-19-infected people have recovered from the COVID-19-related symptoms within one to two months, but some patients were found to have symptoms even after two to three months of COVID infection. The time until symptom resolution appears to depend on premorbid risk factors as well as the severity of the acute illness, and the spectrum of symptoms experienced by the patient. Pre-existing conditions and risk factors are predictors of acute COVID-19 outcomes (such as admission to the intensive care unit and mortality), but the epidemiology of post-COVID-19 syndrome has been less well defined. These long-term manifestations were called as post-COVID-19 symptoms, and it may be the emergence of new symptoms after recovery from acute infection or the persistence of symptoms [4]. The estimated prevalence of post-COVID sequelae is between 10% and 35% [5]. The prevalence and pattern of post-COVID-19 symptoms are questioned. Post-COVID-19 symptoms can be due to ongoing COVID-19 pathophysiological processes. Other than the virological insult, the immune reaction to the virus is also reasonable for these chronic post-COVID-19 symptoms [6]. Despite preliminary guidance from multiple sources, there is not yet a consistent approach to clinical characteristics and risk factors for post-COVID symptoms in patients. Clinical characteristics of the disease will help in appropriate diagnosis, care, public health interventions and policy, and resource planning. Some studies assessed the post-COVID-19 clinical characteristics and their relationship with the severity of COVID-19 infection [7-11], but, to our knowledge, there are fewer studies on post-COVID-19 clinical manifestations in the Indian population. The purpose of this study is to analyze the clinical characteristics of post-COVID-19 symptoms in patients attending a post-COVID-19 clinic who had tested positive for COVID-19 infection.

## Materials and methods

Aims and objectives

Primary

The primary aim of this study is to study the pattern of post-COVID-19 clinical manifestations among patients attending the post-COVID-19 clinic in a tertiary care center in Puducherry, Southern India.

Secondary

The secondary aim of this study is to compare the post-COVID-19 clinical manifestations between hospitalized and non-hospitalized patients.

Ethics approval and consent

The study protocol was approved by the Institute Ethics Committee (IEC), and written and informed consent was obtained from all the participants included in the study.

Study setting and population

The study was conducted in a tertiary care center, Jawaharlal Institute of Postgraduate Medical Education and Research (JIPMER), located in Puducherry, India.

Patients who were coming for post-COVID-19 clinic in a tertiary care center who tested positive for COVID-19 (SARS-CoV-19 RT-PCR positive or rapid antigen positive test positive or radiological diagnosis of SARS-CoV-19 (COVID-19 Reporting and Data System [CO-RADS] 5) [12] were included in this study.

Exclusion criteria

The study excluded COVID-19 survivors with a history of psychiatric illness.

Study design and data collection

We conducted a hospital-based descriptive study. A structured questionnaire was used to collect data regarding socio-demographics, comorbidities, clinical features, and information about acute COVID-19 infections of the participants.

Study methodology

After getting clearance from the Postgraduate Research Monitoring Committee and the Institute Ethics Committee, all patients who attended the post-COVID-19 clinic in the Departments of General Medicine and Pulmonary Medicine, JIPMER, were approached for the study. Patients were included if they had confirmed COVID-19 disease in a hospital-based registry (positive COVID-19 by RT-PCR or RAT, or presumed presence of COVID-19 based on clinical and radiological criteria (CO-RADS 5). Consenting participants filled out the informed consent proforma. Each of the participants was approached in person with a structured proforma containing demographic data, COVID-19 status, other comorbidities, and post-COVID-19 manifestations. The proportion of people developing post-COVID-19 manifestations was noted. Symptoms in the post-COVID-19 period and their relationship with the severity of COVID-19 infection were analyzed. The difference in post-COVID-19 symptoms between hospitalized and non-hospitalized patients was studied. All patients who came to the post-COVID-19 clinic were assessed for symptoms, underwent necessary evaluation, and were treated accordingly.

Post-COVID-19 or long COVID-19 can be divided into two stages: post-acute COVID-19 and chronic COVID. Post-acute COVID-19 is characterized by symptoms extending beyond 3 weeks but less than 12 weeks, whereas chronic COVID-19 has symptoms extending beyond 12 weeks [5]. In our study, this definition was used for recruiting patients, even though multiple definitions are upcoming in multiple trials.

“Acute COVID-19 infection is divided into mild, moderate, and severe according to clinical features and signs. A mild disease is characterized by upper respiratory tract symptoms without shortness of breath or hypoxia. A moderate disease has one of the following: respiratory rate ≥ 24 or SPO_2_ ≤ 93% on room air. Severe disease is characterized by respiratory rate ≥ 30 or SPO_2_ ≤ 90% on room air” [13].

Sample size

The sample size was calculated using OpenEpi Version 3.01 using the formula for sample size for proportions. With the estimated prevalence of post-COVID-19 symptoms to be 82%, as reported by Tomar et al., and 5% absolute precision, the estimated sample size was 227 [14].

Statistical analysis

The statistical analysis was conducted using SPSS Version 19.0 (IBM Corp., Armonk, NY) [15]. Baseline demographic characteristics and clinical characteristics of the patients were presented by descriptive statistics. The normality of continuous data (age, duration) was assessed using the Kolmogorov-Smirnov test. The continuous variables, which are normally distributed, were described by mean ± standard deviation, and the median and interquartile range were used otherwise. Categorical data (clinical characteristics and symptoms) were described using percentages and frequencies and were compared between hospitalized and non-hospitalized patients using the chi-square test. A 95% confidence interval was calculated and reported for the outcome measures. Statistical analysis was carried out at a 5% level of significance, and p<0.05 was considered as statistically significant.

## Results

The present study was conducted in the Department of Pulmonary Medicine post-COVID clinic during the period from August 2021 to May 2022. We have recruited 227 post-COVID patients. The baseline socio-demographic and comorbidities are presented in Table 1.

**Table 1:** Baseline characteristics of study population (N=227)

Variables	Total, n (%)	n (%)	
		Male	Female
Age			
10-19	5 (2.2)	3 (60.0)	2 (40.0)
20-29	22 (9.6)	13 (59.1)	9 (40.9)
30-39	36 (15.8)	19 (52.7)	17 (47.2)
40-49	39 (17.2)	22 (56.4)	17 (43.5)
50-59	72 (31.7)	42 (58.3)	30 (41.6)
60-69	39 (17.2)	19 (48.7)	20 (51.2)
70-79	14 (6.1)	7 (50)	7 (50)
Total	227	126 (55.5)	101 (44.5)
Comorbidities			
Diabetes mellitus	84 (37.0)	51 (60.7)	33 (39.2)
Systemic hypertension	70 (30.8)	40 (57.1)	30 (42.9)
Cardiac disease	15 (6.6)	7 (46.6)	8 (53.4)
Bronchial asthma	14 (6.1)	4 (28.5)	10 (71.4)
Hypothyroidism	8 (3.5)	2 (25.0)	6 (75.0)
Chronic obstructive pulmonary disease	6 (2.6)	4 (66.6)	2 (33.3)
Kidney disease	3 (1.3)	1 (33.3)	2 (66.6)
Addiction history			
Smoking	38 (16.7)	35 (92.1)	3 (7.9)
Alcoholic	37 (16.3)	35 (94.5)	2 (5.5)
BMI (kg/m^2^) (WHO Asia-Pacific BMI classification)			
<18.5 (underweight)	12 (5.2)	7 (58.3)	5 (41.7)
18.5-22.9 (normal)	66 (29)	32 (48.5)	34 (51.5)
23-24.9 (overweight)	52 (22)	35 (67.3)	17 (32.7)
25-29.9 (obese I)	81 (35.6)	45 (55.6)	36 (44.4)
>30 (obese II)	16 (7.04)	9 (56.2)	7 (43.8)

The mean age of presentation of our patients was 48.92+2.22 years. The lower limit of age in our study was 12 years. Among 227 populations, male patients were more in our study constituting 55.50%, whereas female constituted 44.5%. The most common co-morbidity with which the patient presented was diabetes mellitus (84%), followed by systemic hypertension (70%) and cardiac disease (15%). Overall, 16% population had an addiction history in our population. Among our 227 patients, most (81%) of the patients were in the obese category according to the WHO Asia-Pacific BMI classification.

Patient’s clinical features of acute COVID-19 infections, the severity of COVID-19 infection, mode of oxygen delivery, treatment, and COVID vaccination are presented in Table 2.

**Table 2:** Clinical features, severity, treatment, and oxygen requirement during acute COVID infection Note: Proportion in each category was presented; therefore, the total will not add up to 227 (100%). ICU, intensive care unit; NRBM, non-rebreather mask

COVID-19 symptoms	n (%)	Males, n (%)	Females, n (%)
Cough	182 (80.1)	101 (55.5)	81 (44.5)
Fever	137 (60.35)	79 (57.6)	58 (42.3)
Shortness of breath	129 (56.8)	74 (57.4)	55 (42.6)
Loss of smell	27 (11.9)	13 (48.1)	14 (51.8)
Diarrhea	24 (10.5)	13 (54.2)	11 (45.8)
Myalgia	18 (7.9)	11 (61.1)	7 (38.9)
Headache	15 (6.6)	7 (46.7)	8 (53.3)
Chest pain	6 (2.6)	1 (16.7)	5 (83.3)
Loss of appetite	5 (2.2)	3 (60.0)	2 (40.0)
Duration of post-COVID-19 symptoms after acute COVID-19 infections (months)			
1	52 (22.9)	32 (25.3)	20 (19.8)
2	89 (39.2)	49 (38.9)	40 (39.6)
3	34 (14.9)	20 (15.8)	14 (13.8)
4	12 (5.2)	7 (5.5)	5 (4.9)
5	11 (4.8)	5 (3.9)	6 (5.9)
6	15 (6.6)	5 (3.9)	10 (9.9)
7	7 (3.0)	3 (2.3)	4 (3.9)
9	3 (1.3)	2 (1.5)	1 (0.1)
12	3 (1.3)	2 (1.5)	1 (0.1)
14	1 (0.4)	0 (0)	1 (0.1)
Place of COVID-19 treatment			
Home isolation	85 (37.4)	42 (49.4)	43 (50.6)
Ward	107 (47)	66 (61.7)	41 (38.3)
ICU	35 (15.4)	18 (51.4)	17 (48.6)
COVID severity			
Mild	116 (51.1)	64 (55.2)	52 (44.8)
Moderate	40 (17.6)	24 (60.0)	16 (40.0)
Severe	71 (31.2)	38 (53.5)	33 (46.5)
O_2_ therapy			
Nasal prongs	27 (32.5)	16 (59.2)	11 (40.7)
Face mask and NRBM	36 (43.3)	23 (63.9)	13 (36.1)
Noninvasive ventilation	17 (20.6)	9 (52.9)	8 (47.1)
Invasive ventilation	3 (3.6)	3 (100.0)	0 (0.0)
Treatment			
Steroids	109 (48.0)	65 (59.6)	44 (40.4)
Antibiotics	80 (35.2)	51 (63.7)	29 (36.3)
Remdesivir	27 (11.9)	14 (51.9)	13 (48.1)
Baricitinib	7 (3.1)	3 (42.9)	4 (57.1)
Tocilizumab	1 (0.4)	0	1 (100.0)

In our study population, most of the patients were treated in a ward (47%). Most of them had mild severity of acute COVID-19 infection (51.1%). Among treatments, many patients were treated with steroids (48%). Among our study population, most of them used oxygen by face mask and non-rebreather mask (NRBM) (43.3%).

Our study's main aim is to characterize the clinical manifestation of post-COVID-19 who attended the post-COVID-19 clinic presented in Table 3. Among 227 patients, the most common symptom was breathlessness (72%) followed by cough (48%), fatigue (42.3%), and chest pain (14.5%). Most of the patients had normal vesicular breath sounds (68.7%).

**Table 3:** Distribution of symptoms among post-COVID-19 patients Multiple responses present

Symptoms	n (%)	Males, n (%)	Females, n (%)
Breathlessness	164 (72.2)	86 (52.4)	78 (47.6)
Cough	109 (48.0)	63 (57.8)	46 (42.2)
Fatigue	96 (42.3)	57 (59.4)	39 (40.6)
Chest pain	33 (14.5)	21 (63.3)	12 (36.3)
Myalgia	28 (12.3)	13 (46.4)	15 (53.7)
Hemoptysis	21 (9.2)	14 (66.7)	7 (33.3)
Fever	18 (7.9)	11 (61.1)	7 (38.9)
Hair loss	12 (5.3)	4 (33.3)	8 (66.7)
Palpitation	8 (3.5)	5 (62.5)	3 (37.5)
Sore throat	6 (2.6)	5 (83.3)	1 (16.7)
Loss of smell	6 (2.6)	6 (100.0)	0 (0.0)
Sneezing	5 (2.2)	3 (60.0)	2 (40.0)
Headache	4 (1.7)	3 (75.0)	1 (25.0)
Sleep disturbances	3 (1.3)	0 (0.0)	3 (100.0)
Physical examination findings			
Crepitations	54 (23.7)	28 (51.9)	26 (48.1)
Normal vesicular	156 (68.7)	88 (56.4)	68 (43.6)
Wheezing	17 (7.4)	10 (58.8)	7 (41.2)

Among 227 patients, post-COVID-19 clinical manifestations were compared between hospitalized and non-hospitalized patients. Breathlessness was more common in both groups. Post-COVID symptoms were more prevalent among hospitalized patients. However, this was not found to be statistically significant (p<0.05). In our study population, 184 patients were not vaccinated for COVID and 43 patients were vaccinated before acute COVID-19 infection. Only statistical significance was found in palpitation between vaccinated and not vaccinated patients but not found in other symptoms (Table 4).

**Table 4:** Association of post covid symptoms with hospitalization during acute COVID-19 infection and COVID-19 vaccination before acute COVID-19 infection

Post-COVID-19 symptoms	Hospitalized patients (N=142), n (%)	Non-hospitalized patients (N=85), n (%)	P-value
Breathlessness	102 (71.8)	62 (72.9)	0.85
Cough	71 (50.0)	38 (44.7)	0.43
Fatigue	62 (43.7)	34 (40.0)	0.58
Chest pain	23 (16.2)	10 (11.8)	0.35
Myalgia	21 (14.8)	7 (8.2)	0.14
Hemoptysis	12 (8.5)	9 (10.6)	0.59
Fever	10 (7.0)	8 (9.4)	0.52
Hair loss	5 (3.5)	7 (8.2)	0.13
Palpitation	7 (4.9)	1 (1.2)	0.26
Sore throat	4 (2.8)	2 (2.4)	1.00
Loss of smell	4 (2.8)	2 (2.4)	1.0
Sneezing	2 (1.4)	3 (3.5)	0.36
Headache	3 (2.1)	1 (1.2)	1.0
Sleep disturbances	2 (1.4)	1 (1.2)	1.0
Post-COVID-19 symptoms	Pre-COVID vaccinated patients (N=43), n (%)	Not vaccinated patients (N=184), n (%)	P-value
Breathlessness	27 (62.8)	137 (74.5)	0.12
Cough	16 (37.2)	93 (50.5)	0.11
Fatigue	15 (34.9)	81 (44.0)	0.27
Chest pain	4 (9.3)	29 (15.8)	0.27
Myalgia	8 (28.6)	20 (10.9)	0.16
Hemoptysis	1 (2.3)	20 (10.9)	0.13
Fever	1 (2.3)	17 (9.2)	0.20
Hair loss	2 (4.7)	10 (5.4)	1.00
Palpitation	5 (11.6)	3 (1.6)	0.007
Sore throat	1 (2.3)	5 (2.7)	1.00
Loss of smell	3 (7.0)	3 (1.6)	0.08
Sneezing	2 (4.7)	3 (1.6)	0.24
Headache	2 (4.7)	2 (1.1)	0.16
Sleep disturbances	1 (2.3)	2 (1.1)	0.46

Among 227 subjects, the association between the degree of acute COVID-19 severity and post-COVID symptoms was analyzed. Of these, breathlessness (p <0.001) and fatigue (p=0.01) were statistically significant (Table 5).

**Table 5:** Relationship of post-COVID-19 symptoms with acute COVID-19 infection severity

Post-COVID-19 symptoms	Mild, N=116), n (%)	Moderate (N=40, n (%)	Severe N=71, n (%)	P-value	
Breathlessness	69 (59.5)	30 (75.0)	65 (91.5)	<0.001	
Cough	53 (45.7)	17 (42.5)	39 (54.9)	0.35	
Fatigue	42 (36.2)	25 (62.5)	29 (40.8)	0.01	
Chest pain	15 (12.9)	6 (15.0)	12 (16.9)	0.75	
Myalgia	13 (11.2)	9 (22.5)	6 (8.5)	0.11	
Hemoptysis	11 (9.5)	5 (12.5)	5 (7.0)	0.61	
Fever	10 (8.6)	4 (10.0)	4 (5.6)	0 .60	
Hair loss	7 (6.0)	1 (2.5)	4 (5.6)	0.85	
Palpitation	4 (3.4)	1 (2.5)	3 (4.2)	1.00	
Sore throat	4 (3.4)	1 (2.5)	1 (1.4)	0.85	
Loss of smell	3 (2.6)	1 (2.5)	2 (2.8)	1.00	
Sneezing	4 (3.4)	0 (0.0)	1 (1.4)	0.58	
Headache	3 (2.6)	1 (2.5)	0 (0.0)	0.39	
Sleep disturbances	3 (2.6)	0 (0.0)	0 (0.0)	0.29	

## Discussion

Since 2019, the COVID-19 pandemic has resulted in much mortality and morbidity. Even though the literature on the clinical features, severity, treatment, and prognosis of SARS-CoV-2 is abundant, the clinical characteristics of post-COVID-19 patients are not well known when compared to those of acute COVID-19 infection. Post-COVID-19 symptoms affect the patient's quality of life and also delay in attaining baseline health. From the pattern of post-COVID-19 symptoms, rehabilitation has to be planned for improving the quality of life [16]. This is important because post-COVID-19 symptoms make an additional burden on the healthcare system, the patient’s family, and society. Our study was a descriptive study conducted among 227 patients who attended the post-COVID-19 clinic in the Departments of Pulmonary Medicine and General Medicine from August 2021 to May 2022.

The median (interquartile range) age of presentation was 52 (38-59) years, of which males were more (126 patients) compared to females (101 patients) in multiple studies [17,18].

In contrast to our study, females were more common in other studies [19-21]. Males patients were more in our study, which can be due to females being less accessible to the healthcare setting from rural areas and being used to hide their symptoms than males. The dysregulated immunological response also may play a role in this.

Diabetes mellitus (37%) was the most common comorbidity followed by systemic hypertension (30.8%). The same was reported by Budhiraja et al. [22], Garrigues et al. [23], Sykes et al. [24], and Fatima et al. [25]. But in the studies by Halpin et al. [26], Peghin et al. [27], Iqbal et al. [28], and Todt et al. [18], the prevalence of systemic hypertension was more compared to type 2 diabetes mellitus. Drugs used in acute COVID-19 infection and stress during COVID-19 infection may be the reason for diabetes mellitus being the more common comorbidity in our study; however, not all patients' diabetes could be attributed to COVID-19 infection.

In our study patients, 89 (39.2%) presented to the post-COVID-19 clinic after two months of COVID-19 positivity. The different duration was mentioned in various studies. This may be due to different definitions or periods of disease resolution stage.

A total of 85 (37.4%) patients were isolated at home, 107 (47%) patients were admitted to the ward, and 35 (15.4%) patients were admitted to the intensive care unit during their acute COVID-19 infection. In our study population, breathlessness (72.2%) was the most common clinical symptom followed by cough (48%) and fatigue (42.3%) in contrast to Sigfrid et al.’s study in which fatigue was the most common symptom [29]. Even though mild (51.1%) severity of acute COVID-19 infection was more common, as reported in other studies, the severe COVID-19 infection (31.2%) proportion was also high in our study compared to other studies; hence, further detailed evaluation is needed to know the role of severity of acute COVID-19 infection in post-COVID-19 [30]. We found that the frequency of post-COVID-19 symptoms was similar in both hospitalized and non-hospitalized patients. However, the site of treatment may vary as studies adopted different guidelines for management. In our study, the patient was managed according to the Indian Council of Medical Research (ICMR) guidelines, based on clinical features, vitals, and the need for oxygen.

Strengths

The defined duration between acute COVID-19 infection and presentation of post-COVID-19 symptoms was elucidated in our study. This is the first study on the clinical characterization of post-COVID-19 symptoms in southern India. Our study emphasizes the prevalence of post-COVID-19 symptoms in hospitalized and non-hospitalized patients.

Limitations

As this is a single-center study, the results cannot be generalized. In this study, patients were not followed; hence, final diagnosis and management were not mentioned. The definition of post-COVID-19 is an evolving one; hence, the definition used in our study differs from the recent WHO one [5].

Future directions

Our study assessed only clinical characteristics of post-COVID-19 manifestations; hence, further evaluation and treatment have to be studied depending on the symptoms pattern. we assume that this study proceeds as an initial point to develop a multidisciplinary team for the diagnosis and management of post-COVID-19 symptoms. Post-COVID-19 symptoms may be due to abnormal immune response; hence, further studies are needed to evaluate the role of cytokines and immune dysregulation in post-COVID-19 symptoms, and the burden of post-COVID-19 and its relationship with their risk factors has to be explored.

## Conclusions

The most common post-COVID-19 symptom in our population was breathlessness (72.2%), followed by cough (48%), fatigue (42.3%), and chest pain (14.5%). Hence, a generalized approach is needed with a specialized approach for each symptom. While comparing post-COVID-19 symptoms, a similar frequency of symptoms was found between hospitalized and non-hospitalized patients. Among post-COVID-19 symptoms, breathlessness and fatigue had a significant relationship with the severity of acute COVID-19 infection. This is a single-center subjective study in which post-COVID-19 clinical symptoms were characterized and described. Further studies are needed to evaluate the symptoms objectively by imaging and pulmonary function test, quality of life using questionnaires, and the need for pulmonary rehabilitation in post-COVID-19 patients.
